# Risk Factors for Brachial Plexus Birth Injury

**DOI:** 10.3390/children5040046

**Published:** 2018-03-29

**Authors:** Emily Louden, Michael Marcotte, Charles Mehlman, William Lippert, Bin Huang, Andrea Paulson

**Affiliations:** 1 Division of Pediatric Orthopaedic Surgery, Cincinnati Children’s Hospital Medical Center, Cincinnati, OH 45229, USA; emily.louden@cchmc.org (E.L.); charles.mehlman@cchmc.org (C.M.); william_lippert@hotmail.com (W.L.); 2 Good Samaritan Hospital, Department of Obstetrics and Gynecology, Division of Maternal/Fetal Medicine, Cincinnati, OH 45229, USA; michael_marcotte@trihealth.com; 3 Division of Biostatistics and Epidemiology, Cincinnati Children’s Hospital Medical Center, Cincinnati, OH 45229, USA; Bin.Huang@cchmc.org; 4 Division of Physical Medicine and Rehab, Cincinnati Children’s Hospital Medical Center, Cincinnati, OH 45229, USA

**Keywords:** brachial plexus birth injury, oxytocin, uterine tachysystole, risk factors

## Abstract

Over the course of decades, the incidence of brachial plexus birth injury (BPBI) has increased despite advances in healthcare which would seem to assist in decreasing the rate. The aim of this study is to identify previously unknown risk factors for BPBI and the risk factors with potential to guide preventative measures. A case control study of 52 mothers who had delivered a child with a BPBI injury and 132 mothers who had delivered without BPBI injury was conducted. Univariate, multivariable and logistic regressions identified risk factors and their combinations. The odds of BPBI were 2.5 times higher when oxytocin was used and 3.7 times higher when tachysystole occurred. The odds of BPBI injury are increased when tachysystole and oxytocin occur during the mother’s labor. Logistic regression identified a higher risk for BPBI when more than three of the following variables (>30 lbs gained during the pregnancy, stage 2 labor >61.5 min, mother’s age >26.4 years, tachysystole, or fetal malpresentation) were present in any combination.

## 1. Introduction

For every 1000 live births, 0.5 to 3 brachial plexus birth injuries occur in the United States [1,2]. Nearly thirty percent of those with brachial plexus birth injury (BPBI) have permanent neurological deficits or impairments [1,2]. Over the course of decades, fluctuating rates of BPBI have been reported despite advances in care that would otherwise be expected to decrease the incidence of BPBI [2]. This highlights the incomplete understanding of the risk factors associated with this injury. There are many commonly accepted risks that have sound statistical backing, yet gaps in our understanding remain. Among babies who have sustained BPBI, only half had known risk factors [1,3]. According to Foad et al., there are still many questions surrounding the etiology of a BPBI. Factors presented include gestational diabetes, fetal macrosomia, instrument-assisted delivery, prolonged labor and/or breech delivery [1]. Infants are often born with one or more of the previous listed risk factors [1]. According to Volpe et al., increased birth weight and gestational diabetes were associated with an increased risk of BPBI when compared to babies born with a birthweight less than 3499 g [4]. The purpose of this study was to search for previously unidentified risk factors or combinations of risk factors. Secondarily, we aimed to identify those risk factors that are modifiable, with the intent to determine potential preventative measures that could result in a decreased incidence of BPBI injuries.

## 2. Materials and Methods

### 2.1. Study Population

Once the institutional review board, through Cincinnati Children’s Hospital Medical Center, gave their approval (approval no. 2010-1242), a list of all mothers who delivered between 2005 and 2010 at a large tertiary care institution was obtained. A convenience sample of 50 mothers who gave birth to a child with a BPBI (cases) were randomly selected based on the International Classification of Diseases 9 (ICD9) code for brachial plexus birth injury (767.6) within the medical records. The database utilized to determine controls without BPBI included 18,973 deliveries taking place from 2005–2011. Each was randomized twice. First, a random number was assigned, and then the random number function in Excel was utilized to determine the 150 controls. Twins represented four controls, while all others were singleton births. BPBI was determined at time of birth by the pediatrician attending the delivery, based on a clinical exam. Identified cases were not followed after discharge to determine their functional outcomes.

Upon further review of hospital records, 132 controls and 52 cases were included in the final analysis. Two controls were misclassified and actually resulted in a BPBI so were moved to cases, bringing the case total to 52 as a convenience sample. Initially 150 controls were selected, but with the two controls being misclassified, this brought the total to 148. Of the 148 remaining controls, 18 had significant data missing, including maternal weight gain or no labor slips used to evaluate tachysystole, resulting in 132 controls.

### 2.2. Data Collection

Data was retrospectively collected on antepartum, intrapartum, and postpartum variables. Excessive weight gain by mother was defined as >30 lbs gained throughout the gestational period. Persistent fetal malpresentation was defined as occiput posterior, occiput transverse, rotation with forceps or vacuum, breech birth, or version for second twin. Tachysystole was defined using the National Institute of Child Health and Human Development criteria as greater than five contractions in 10 min period, averaged over 30 min [4]. Macrosomia was defined as >4500 g. Trained labor and delivery nursing personnel at the birthing hospital reviewing monitoring strips to determine the number of labor contractions and whether tachysystole was present or not included in the stage of delivery at that time. Each slip was reviewed by one nurse without measurement of intra- or inter-rate reliability.

### 2.3. Statistical Analysis

Analysis was conducted using Statistical Analysis Software (v.9.2, SAS Inc., Cary, NC, USA) and SOLAS (v.4.0, Statistical Solutions Ltd., Cork, Ireland) for Missing Data Analysis and R (Multivariate Imputation by Chained Equations) MICE [5]. Univariate analysis provided raw odds ratio (OR) estimates, 95% confidence interval (CI) and two-tailed *p*-values, with <0.05 considered statistically significant. Multivariable logistic regression was used stepwise to identify the most important risk factors. All significant variables were eligible to be included and dichotomized before entering into the initial model. For predictive variables that continued to be significant, Classification and Regression Tree (CART) analyses determined the best cut point with both forward and backward procedures and those significant after the adjustment with other risks remained.

Due to missing data, we conducted a multiple missing data imputation (MI) with MICE. Five repeated sets of missing data were imputed; the analyses results were combined taking account of the between and within imputation variations. The results from two sets of MI technique and from complete case analyses were consistent; therefore, complete case results were reported. The results of the final multivariable logistic regression analyses included adjusted OR, 95% CI, *p*-values, C-statistics and an Receiver Operating Characteristic (ROC) curve; predicted probability for possible combinations of the risks, best sensitivity and specificity were identified by the shortest distance method.

## 3. Results

The average maternal age of the cases was 27.8 years and the average age of the controls was 26.2 years. The average gestational age was 38.7 weeks for the cases and 37.4 weeks for the controls (Table 1). Table 1 illustrates the many characteristics of maternal and infant factors for the control and case groups. As shown, the body mass index (BMI) at the beginning of the pregnancy was 26.62 for cases and 26.06 for controls. Excessive weight gain was observed in 71% of cases compared to 52% of controls. In addition, the infant birth weight was 3817.44 g for cases and 2987.15 g for the controls. Six percent of the cases and 11% of controls were delivered via cesarean section; the remaining had vaginal or operative vaginal delivery.

Comparison results between risk factors for BPBI controls and case are exemplified. The univariate analysis reconfirmed many already established risk factors, such as shoulder dystocia (78.8, OR 95% CI: 21.9–283.7; *p* < 0.0001), macrosomia (24.4, OR 95% CI: 1.3–461.7; *p* = 0.012), gestational diabetes (4.1, OR 95% CI: 1.4–12.2; *p* = 0.0073), persistent fetal head malpresentation (OR 95% CI: 1.2–6.3; *p* = 0.0119). Oxytocin was used in 79% of cases and 60% of controls. Mothers who received oxytocin were 2.5 times more likely to have an infant with a BPBI injury than those who did not (OR 95% CI: 1.2–5.4; *p* = 0.0135). Tachysystole was present in 74% of cases and 44% of controls (Table 2). The odds of a BPBI injury occurring was 3.7 times higher for those who experienced tachysystole (OR 95% CI: 1.7–7.9; *p* = 0.0006) (Table 2).

With the CART analysis, the age of the mother was dichotomized at 26.4 years of age and 61.5 min for stage two length of labor. Based on all possible combination of variables, the logistic regression predicted the probability for each case, ranging from 3.3% with none of the five risks being present, to 95.2% with all five variables present. The use of MICE allowed for a stronger technique to account for the non-monotone patterns and randomness of missing data over the single regression model missing data imputation. MICE also helped with inputting plausible data valves for the missing data.

Table 3 illustrates the logistic regression summary statistics with the predicted calculated probability based on all combinations of the five variables using combined imputed data sets with MICE. Four variables showed significance: persistent fetal head malposition (0.25, 2.45), tachysystole (0.33, 2.19), mother’s age (0.61, 2.39), and stage 2 labor length greater than 61.5 min (1.37, 3.37). The last variable—maternal weight gain—did not show significance. (Table 3).

Logistic regression of the combined imputed data sets of the five variables and the initial non-missing data input results were very similar. The range of predicted probability of BPBI was 1.9% risk without any of the five variables being present to 95.4% risk with all five variables being present. The best cut point was identified as 0.32, which corresponds to the presence of three or more risk variables, indicating the threshold from a lowered risk to an increased risk. According to the confidence interval, excessive maternal weight gain was not significant at 0.83. Table 4 clarifies the vairables of the mutliple logistic regression model showing the predicted calculated probabilty for each case without data imputation, which showed significance within three variables: persistent fetal malpresentation, tachysystole and mother’s age. Using this cut point over the five data sets, we estimate the average area under the curve to be 0.85, with 71% sensitivity and 81% specificity. (Table 4).

## 4. Discussion

The severity of BPBI ranges from mild nerve stretch injuries with rapid recovery to nerve root avulsions with no spontaneous recovery. [1] Many infants with BPBI recover with minimal or no residual functional deficits within the first 6 months of life. [2] However, 20% of children do not fully recover [1,6]. A significant percentage of BPBI patients have functional limitations, bony deformities, or joint contractures [6]. The lasting effects of BPBI may lead to lifelong musculoskeletal functional implications. [6] Treatment of these patients is complex and requires a multidisciplinary approach [6]. Prevention of BPBI is paramount to treatment of its residual sequelae.

Our study identified the occurrence of tachysystole as a risk factor for BPBI. Oxytocin is frequently given to laboring women to speed uterine contractions and shorten labor, but a high dose can cause the uterus to contract too quickly and cause tachysystole and fetal distress [7]. Despite being such a common treatment, the ideal dose is still unknown [7,8]. These two risk factors are clearly interrelated, as tachysystole is the most common adverse side effect associated with the use of oxytocin [9]. It is uncertain if both are risk factors alone, or only when used together, given the co-dependence of these two variables.

The use of oxytocin has received a great amount of attention in recent years. At least one group of researchers hinted at an association between BPBI and oxytocin-augmented labor, but it was not determined to be statistically significant. [10] Oxytocin use has been the focus of quality improvement and risk management in obstetrical care and safety [11,12,13]. Prevention of tachysystole is cited as a nursing goal in the care of laboring mothers [14]. Our findings coupled with these cited issues support the need for further research into the safe management of oxytocin dosing and tachysystole prevention.

Several previously identified predictors of BPBI were revalidated by our study. Shoulder dystocia, macrosomia, gestational diabetes, fetal malpresentation, and excessive maternal weight gain have all previously been noted as risk factors [1,2,11,15,16,17,18,19]. Although statistically sound, most of these risk factors offer little to no potential to aid the obstetrician in attempting to prevent BPBI during delivery [20]. Predictive values do not come from having a commonly-occurring risk factor associated with a rare birth outcome. While oxytocin administration and tachysystole during labor showed associations with increased incidence of BPBI injury, these two risk factors alone do not warrant a change in clinical care to prevent potential BPBI injury. Moreover, some risk factors are not identifiable or easily assessed during prenatal care or intrapartum labor. In a study by Blickstein et. al., only 3 infants were diagnosed with brachial plexus birth injury and 27 with shoulder dystocia out of 236 vaginally delivered neonates weighing at least 4200 g [21]. Studies have cited a significantly higher rate of macrosomic infants yet yielded less incidence of BPBI and shoulder dystocia [22,23,24]. Clinical estimates and ultrasound biometry as methods for obtaining an antenatal estimate of birth weight have often been found to be inaccurate, especially for extremely large or small infants [25,26,27].

It was interesting to note the low rate of macrosomia in our study—0/131 within the control group and 4/52 within the case group. Different definitions have been used in the past, including a 4000 g threshold, but the current American College of Obstetricians and Gynecologists definition is a birth weight >4500 g [22]. There were only 7.7% (4/52) of macrosomic babies in the case group and no macrosomic babies in our control group. Macrosomia is a well-established risk factor for BPBI with the incidence increased by as much as fourteen-fold [16,19,21]. We recognize that ultrasound is an imperfect predictor of macrosomia, macrosomia is an imperfect predictor of shoulder dystocia, and shoulder dystocia is an imperfect predictor of BPBI [24,25,26]. This is also coupled with the paradox that while rates of macrosomia have been decreasing, rates of shoulder dystocia have been increasing [27].

Birth-related fetal injuries range from fractured bones (clavicle, humerus shaft), which have little to no long-term significance, to BPBI, to varying degrees of anoxic brain injury, or fetal demise [2]. A substantial portion of prenatal care is focused on trying to prevent fetal macrosomia by minimizing abnormal maternal weight gain and managing gestational diabetes [28]. According to Institution of Medicine (IOM) recommendations, excessive weight gain during pregnancy for overweight women is defined as a gestational weight gain (GWG) greater than 30 lbs [29,30,31]. The overall BMIs of women in the case group (26.05 kg/m^2^) and control group (26.62kg/m^2^) placed them in the overweight BMI category. [29,30,31]. The mean weight GWG was 44.01 lbs for the women in the control group and 43.38 lbs for women in the case group which validates GWG as a modifiable risk factor which can be monitored throughout the pregnancy.

In order to be a clinically useful predictor of BPBI injury, the risk must be accurately ascertained and assessed at a time when an intervention is still feasible. The continuous variables of weight gain during pregnancy (>30 lbs) and mother’s age (>26.4 years) can easily be identified by the clinician prior to the birth. The other three variables in the model—fetal malpresentation, length of the second stage of labor (dilation to delivery >61.5 min) and tachysystole—can be observed, monitored, and potentially corrected by the clinician through an intervention prior to the delivery. These five variables have each been individually identified but independently provide little prognostic guidance for clinical care. Nonetheless, when combined, they have a strong sensitivity and specificity—71% and 81%, respectively. The baby is at an increased risk of sustaining a BPBI, when the mother has three or more of the variables. The combination of these risks could identify a population of laboring women in which a change in treatment during labor and delivery may be warranted to further minimize BPBI. In addition, long term follow-ups would also be warranted to help change the incidence of BPBI.

The study’s limitation of most significance was missing data. Along with this, the primary focus was on the birth and risk factors and not the functional outcomes following the BPBI. All retrospective BPBI babies were identified at the time of birth by the pediatrician; however, clinically, all BPBI are not immediately recognized until a follow-up with their pediatrician. In addition, cesarean section was not discussed with respect to validity due to the limited number of cases and controls. Another limitation in the study was the selection of 30 lbs as the definition of excessive weight; according to IOM, excessive maternal weight gain for women with a BMI over 24.99 is defined as weight gain of more than 25 lbs. Despite these limitations, confidence was given to the study’s results, due to the finding that our logistic regression without and with imputation was comparable. Additional research in this area along with use of oxytocin effects on macrosomia is warranted.

## 5. Conclusions

This case control study indicates that when three or more of the following variables were present in any combination, a higher risk for giving birth to an infant with BPBI is created: weight gain during pregnancy, longer than 61.5 min of stage 2 labor, >26.4 years of age, tachysystole, or persistent fetal malpresentation. These findings are the first of their kind, but a prospectively validated study on modifiable risk factors is warranted to determine if a reduction of BPBI injuries results.

## Figures and Tables

**Table 1:** Characteristics of maternal and infant factors for controls and cases.

Variable	Control Mean (SD)	Cases Mean (SD)	*p*-Value
Maternal age (months)	314.8 (80.6)	333.6 (73.42)	0.1444
Gestational age (weeks)	37.42 (3.51)	38.69 (1.38)	0.0122 *
Number of contractions while in labor	130.55 (121.74)	199.74 (139.46)	0.0029 *
Maternal weight gain (lbs)	44.01 (14.42)	43.38 (10.05)	0.8265
BMI at beginning of pregnancy	26.05 (7.68)	26.62 (5.74)	0.6521
BMI at the end of pregnancy	31.35 (8.25)	31.63 (8.36)	0.8467
One hour glucola screening test	95.43 (36.65)	118.82 (28.95)	0.0014 *
Length of oxytocin administration (min)	372.47 (247.24)	524.15 (291.59)	0.009 *
Length of stage 1 labor (min)	822.36 (3275.35)	459.33 (343.97)	0.4315
Length of stage 2 labor (min)	34.49 (47.87)	93.69 (82.81)	<0.0001 *
Length of stage 3 labor (min)	3.75 (5.67)	4.19 (3.66)	0.6077
Total length of labor (min)	862.3 (3260.86)	556.39 (374.32)	0.5056
One hour post-glucose (mg/dL)	114.79 (33.56)	166.63 (29.75)	0.0017 *
Infant birth weight (g)	2987.15 (746.87)	3817.44 (604.97)	<0.0001 *

Significant values noted as * *p* < 0.05. SD: standard deviation, BMI: body mass index.

**Table 2:** Comparison results between risk factors for brachial plexus birth injury (BPBI) controls and cases.

Categorical	Control	Cases	OR (95% CI)	Chi-Square/Fisher
Excessive maternal weight gain (>30 lbs)	62/119 (52.1%)	31/44 (70.5%)	2.2 (1.05–4.6)	0.0356
Persistent fetal head malpresentation	15/128 (11.7%)	14/52 (26.9%)	2.8 (1.23–6.28)	0.0119
Gestational diabetes	6/123 (4.9%)	9/52 (17.3%)	4.1 (1.37–2.15)	0.0073
Maternal obesity at the beginning of pregnancy	29/119 (24.3%)	10/44 (22.7%)	0.9 (0.4–2.07)	0.8273
Maternal obesity at end of pregnancy	59/122 (48.4%)	27/47 (57.4%)	1.4 (0.73–0.84)	0.2897
Oxytocin administration	78/131 (59.5%)	41/52 (78.9%)	2.5 (1.19–5.37)	0.0135
Tachysystole	38/87 (43.7%)	37/50 (74%)	3.7 (1.71–7.85)	0.0006
Occiput anterior delivery position	106/129 (82.2%)	25/52 (48.1%)	4.9773	0.0001
Epidural administered	117/131 (89.3%)	50/52 (96.2%)	3 (0.66–13.65)	0.2434
Infant clavicle fracture	0/130 (0%)	5/52 (9.6%)	30.2 (1.64–557.01) *	0.0033 *
Infant humerus fracture	0/130 (0%)	2/52 (3.9%)	12.9 (0–273.83) *	0.1610 *
History of difficult delivery	5/80 (6.3%)	3/27 (11.1%)	1.9 (0.42–8.43)	0.4133
History of fetal malpresentation	1/82 (1.2%)	1/27 (3.7%)	3.1 (0.19–51.58)	0.4358
Macrosomia	0/131 (0%)	4/52 (7.7%)	24.4 (1.29–461.7) *	0.0120
Pre-eclampsia	12/127 (9.5%)	1/52 (1.9%)	0.2 (0.02–1.48)	0.1123
Prostaglandin administration	19/126 (15.1%)	5/52 (9.6%)	0.6 (0.21–1.7)	0.4698
Shoulder Dystocia	3/132 (2.2%)	33/51 (64.7%)	78.8 (21.9–283.72)	<0.0001

* OR: odds ratio (95% CI) and *p*-values are calculated by adjusting zero counts (add 0.5).

**Table 3:** Logistic regression summary statistics with the predicted calculated probability based on all combinations of the five variables, using combined imputed data sets (*n* = 5) with MICE.

	Intercept	Persistent Fetal Head Malposition	Tachysystole	Mother’s Age (26.4 years)	Stage 2 Labor Length (>61.5 min)	Excessive Maternal Weight Gain (>30 lbs)
Beta ^1^	−3.944	1.351	1.259	1.503	2.371	0.487
Beta (SD) ^2^	0.638	0.549	0.467	0.451	0.499	0.478
95% CI ^3^	(−5.21–−2.68)	(0.25–2.45)	(0.33–2.19)	(0.61–2.39)	(1.37–3.37)	(−0.47–1.44) *

* Excessive maternal weight gain not significant. ^1^ Beta: logistic regression coefficient estimate (the coefficient tells you how much the dependent variable is expected to increase when that independent variable increases by one, holding all other independent variables constant.). ^2^ Beta (SD): standard deviation estimate for Beta. ^3^ 95% CI: 95% confidence interval for Beta.

**Table 4:** Initial parameter estimation of the multiple logistic regression model showing the predicted calculated probability for each case without missing data imputation.

	Intercept	Persistent Fetal Mal-Presentation	Tachysystole	Mother’s Age (26.4 years)	Stage 2 Labor Length (≥61.5 min)	Excessive Maternal Weight Gain (>30 lbs)
Beta ^1^	−3.3769	0.8892	0.9953	1.6104	2.1181	0.7483
Beta (SD) ^2^	0.7065	0.7262	0.5041	0.5518	0.5479	0.5078
95% CI ^3^	(−4.78–−0.99)	(−0.53–2.31)	(0.01–2.00)	(0.53–2.69)	(1.02–3.21)	(−0.27–1.76)

^1^ Beta: logistic regression coefficient estimate. ^2^ Beta (SD): standard deviation estimate for Beta. ^3^ 95% CI: 95% confidence interval for Beta.
